# Longitudinal Distress among Brazilian University Workers during Pandemics

**DOI:** 10.3390/ijerph18179072

**Published:** 2021-08-27

**Authors:** Murilo Ricardo Zibetti, Fernanda Barcellos Serralta, Chris Evans

**Affiliations:** 1 Post Graduate Program in Psychology, Universidade do Vale do Rio dos Sinos—UNISINOS, São Leopoldo 93022-970, Brazil; fernandaserralta@gmail.com; 2 Department of Psychology, The University of Sheffield, Sheffield S1 1HD, UK; chris@psyctc.org

**Keywords:** COVID-19, pandemics, quarantine, psychological distress, longitudinal studies

## Abstract

The present study aimed to examine changes in mental distress in Brazilian university workers during the pandemic. All workers (*n* ≃ 1850) of an institution were invited to respond to a survey that took place in three stages, with collections in May (*n* = 407), June/July (*n* = 258), and August (*n* = 207) 2020, and included questions on demographic, health, general and psychological support, and psychometric assessment of mental distress (Clinical Outcome Routine Evaluation- CORE-OM) combined with an open question about major concerns. The results of the Multilevel Modeling analysis pointed to the absence of significant differences across the repeated measures of distress. The only variable associated with increased psychological distress over time was a lower level of support for household chores. Qualitative analysis of the reported major concerns was carried out with a sub-sample who showed reliable deterioration in CORE-OM across time (*n* = 17). The diversity of concerns reported by this group reinforced that work–life imbalance contributes to mental distress of university workers during the pandemic. Low response rate, although not unexpected due to the circumstances, limits the generalization of findings. The present data suggest that in addition to issues related to contagion and specific restricted measures to contain the spread of the disease, the personal reorganization of life required to maintain activities at home and work can be an important contributor to pandemic-related psychological distress.

## 1. Introduction

In March 2020, the World Health Organization (WHO), declared the spread of the SARS-CoV-2 virus had reached pandemic levels. The circulation of a highly transmissible virus, potentially lethal and untreated, resulted in the imposition of behavioral norms to avoid contagion [1]. The main one involved social distancing that resulted in extensive home confinement [2]. Although it has varied locally, from instructions to stay at home to compulsory isolation, guidelines for social distancing were adopted by several countries.

It was quickly realized that COVID-19 and the measures to contain it were significant psychological stressors [2]. In addition to the possibility of contagion and the isolation, economic losses, changes in routine, and loss of social support also contributed to increased mental struggle [3]. Therefore, since the beginning of the pandemic, researchers have warned of the impacts on mental health and warned that health systems should be prepared to provide psychosocial support to the general population [2,3,4].

International and national survey studies corroborated these expectations by demonstrating the immediate impacts of the pandemic on mental health in the general population [5]. High rates of mental distress have been identified in cross-sectional studies conducted in countries such as China [6], Italy [7], the United States [8], and Brazil [9,10]. Worsening symptoms such as sadness, anxiety, sleep difficulties, and even suicidal ideation have been reported [6]. A relative consensus was established that belonging to the female gender, being younger, and having history of mental disorder, were risk factors for greater mental distress in this period [6,10,11].

In addition to the immediate mental health effects of the pandemic on the population, some studies have made comparisons before and after social distancing measures. For example, a study conducted in the United Kingdom indicated that the percentage of people classified as suffering from mental illness rose from 19.4% between the years 2017 and 2019 to 30.6% in the first month of isolation [12]. Similar data on deterioration, when compared to those before isolation measures, were obtained in other studies and stages of the pandemic in the United Kingdom [11]. A cross-sectional study conducted in the United States indicated that each day more during the early months of the pandemic predicted an increase of 11% in the odds to change to a higher category of mental distress [8]. These before versus during the pandemic between-group comparison studies presented a picture of psychological deterioration [13]. However, the absence of within-participant follow-up studies creates biases, making conclusions about the evolution of mental health indicators uncertain.

Longitudinal studies of the change in mental health indicators during the pandemic are emerging. A study conducted in China, with an interval of 30 days between the two collections during the pandemic, detected levels of stabilization of mental health indicators at levels below expectations [13]. In Latin America, data collected in Argentina in the first two weeks of the lockdown indicated changes with a small effect size in the indicators of depression, anxiety, and affection [14]. On the other hand, a study conducted in India reported worsening levels of stress, depression, and anxiety over the first two months of lockdown [15]. In Spain, a study carried out between the second and the fifth week of the pandemic indicated complex effects with worsening depression and stabilization of anxiety and post-traumatic stress [16].

Previous studies demonstrate the complexity of the pandemic effects and isolation measures on the general population’s mental health. There are three obvious possibilities: (1) deterioration of mental health with aggravation of stressors over time; (2) stabilization of levels of distress due to the maintenance of conditions; (3) recovery of the initial impact due to the reorganization of the routine, individual resilience, and identification of support networks. A meta-analysis of longitudinal studies demonstrated modest effects on depression and anxiety and the absence of longitudinal effects on most of the investigated psychological variables [17]. This study also noted the great variability of effects reported in these studies. The different results are possibly associated with the different methods (sampling, measurement), forms and needs of fulfilling social isolation [17], personal differences in wealth and resources to survive [18], and the different governmental responses to the containment of the pandemic [19].

Longitudinal data on mental health during the pandemic in Brazil is scarce. The objective of the present study was to describe changing mental struggles during the pandemic in a specific group of Brazilian university workers. Few studies have been conducted with university workers, though they are interesting, having access to scientific information and often being able to move work activities to home. Cross-sectional studies conducted in Italy [20] and Spain [21] have reported high levels of anxiety and depression in the population. In addition to the stressors affecting the general population, university workers have found themselves under a particular pressure to keep up their teaching work and supporting their students, but at a distance [22]. In a previous study, we identified that the concern most mentioned by these professionals involved issues related to work, from the quality of the services provided to the pervasive pressures of remote office working [23]. In the same study, we identified that 98% of these professionals reported complying with the social distance measures.

The present study aimed to build on the earlier report by examining possible changes in psychological distress reported by university workers during the initial period of the pandemic, examining the effect of social isolation measures on this population. Secondary objectives were to verify the variables associated with the increase or decrease in suffering and to identify the concerns of people with a worsening mental health status.

## 2. Materials and Methods

### 2.1. Study Design

It was a longitudinal and mixed design (quantitative and qualitative). The study’s approach was pragmatic as the primary intention of the survey was to provide support to the university community through screening for mental distress and the provision of support measures to these people (remote psychological first aid and referrals). The study used longitudinal data from the three months of the intervention project. This interval was designed based on the evolution of the pandemic’s first wave and the end of the first semester of classes.

### 2.2. Participants

Approximately 1850 university employees or service providers were potentially eligible for research and were invited to participate in the online survey. No inclusion criteria other than working for the university were imposed and there were no exclusion criteria. In the first data collection, in May 2020, 407 responses were received. The participants had a mean age of 37.5 years, and the majority were female (68%) and had been, on average, social distancing for 59 days. In the second stage of data collection, in June, 258 responses were received. These participants had a mean age of 40.9, 72% were female, and they had a mean time in social isolation of 92 days. Finally, 207 participants responded to the third stage in August, who had a mean age of 41 years, 72% were female, and had a mean of 130 days in isolation. Except for the time in isolation, which predictably increased, there were no statistically significant differences (*p* > 0.05) between the proportion by gender and the average age of the participants in the three stages.

The selection criterion for inclusion in this analysis of change was having responded to at least two stages of data collection (*n* = 256). Only 36 participants responded in all three stages, 87 participated in the first and second stages, 70 in the second and third stages, and 63 participants in the first and third stages.

### 2.3. Instruments

An electronic survey was developed for online completion by the participants. The data on this form can be grouped as follows:

General demographic items: mainly focused on the description of the sample, including issues such as age, gender, and position in the institution.
Demographic and self-care items related to the pandemic: developed to assess factors potentially related to mental and physical health in the pandemic. These included the time of isolation, belonging to a risk group for COVID-19, living or being a worker in essential areas, support received, and health habits (food, alcohol consumption, relaxing activities, and exercise) during pandemics. The questionnaire also contained an open question about the main current concerns of the participants. A very detailed description of the measures, used for each of these variables, was presented in a previous publication [23]. In this study, only the significant variables in that previous study were included as predictors of mental health, namely exercise, support for daily household activities and availability of people to listen, and psychological and psychiatric support.
Clinical Outcomes in Routine Evaluation—Outcome Monitoring (CORE-OM) [24,25,26]: this is a self-report questionnaire developed in the United Kingdom for monitoring treatment outcomes in mental health. The original version has 34 items answered in a Likert scale format. The questions of the instrument can be grouped as risk scores (6 items) and non-risk (NR) (28 items). For the present study, we chose to use the non-risk items as this set constitutes an indicator of mental distress. In the original study, the NR scale had excellent indicators of internal consistency (Cronbach’s α = 0.94) [24]. It was used for the present study, the Brazilian Portuguese version adapted by Santana et al. (2015) [27] following the guidance of the CORE System Trust (www.coresystemtrust.org.uk/cst-translation-policy accessed on 12 January 2021). The internal consistency (Cronbach alpha) of the NR was 0.94 for the May stage and 0.93 for both the June/July and August stages.

### 2.4. Procedures

The study was carried out at a university in southern Brazil after an agreement between the researchers and the university managers, specifically from the committee responsible for the pandemic contingency plan. Participation was invited through an institutional email sent to all employees and service providers in the institution. This email contained a link with access to an online form that took about 30 min. The form remained open for approximately 10 days from the invitation and further e-mails encouraging participation were sent during this period.

Data collection was carried out three times between May and August 2020, maintaining an interval of at least 4 weeks between the end of a collection and the beginning of the subsequent one. The first collection took place between the 9th and 10th week after the interruption of on-site activities at the University. The classes and other university activities remained remote throughout the data collection period.

Pseudonymous linkage of repeat completions was based on a code generated by the participant, maintained in the three collections. Participants who allowed further contact on the form received feedback of results from the survey between the stages.

### 2.5. Data Analysis

Firstly, analyses described the sample and checked the internal consistency of the CORE-OM instrument. Then, inferential analyses were performed to ascertain the effect on the mental distress indicator over time. For this analysis, the direct effect of time and, separately, the interaction of the effects with predictors were tested.

To evaluate the effect of time, repeated measures were considered, accounting for the variation between subjects through the Multilevel Modeling (MLM) analysis [28,29,30]. Each of the study stages was categorized as a level, regardless of the time interval between them (i.e., stages one, two, and three). MLM handles non-participation across the three stages by estimating a linear path of any two scores completed. Slope against time was first treated as fixed (stable among participants) then a random effect of time, i.e., different score slopes per participant, were also allowed.

After the identification of changes over time in the NR score, analyses were performed to find out predictors of this change. Stable (gender and age) and time-varying predictors (exercise practice, support in domestic activities, people available to talk, psychological and psychiatric follow-up) were tested separately. Though the latter are time-varying predictors, moderate and high levels of correlation between repeated measures were found (τb coefficient varying from 0.371 to 0.694) and only levels at the first participation were entered. Separate MLM analyses were performed for each of the potential predictors of change. Finally, score changes for participants who answered at least twice were evaluated using the Reliable Change Index (RCI), which indicates, for each participant, whether changes were larger than would be expected 5% of the time based on the reliability of the measure [31,32]. For each of the two time periods, the RCI allowed participants to be categorized as deteriorating, not reliably changing, or improving.. These proportions were compared across the two time periods, and we used this to find participants with deterioration and to inspect qualitative responses provided in the open question about concerns during the pandemic. For this simple analysis, we used the categories previously reported [23] that were identified using the Consensual Qualitative Research for simple qualitative data method—CQR m [33]—in the first stage of study.

### 2.6. Ethical Considerations

The study was designed in compliance with the guidelines on research with human beings in Resolution 466/2012 and in 510/2016 of the National Health Council (Brazil, 2012; 2016) and Resolution 016/200 of the Federal Council of Psychology (2000). The project was approved by the Ethics Committee of the university where the research was carried out (CAAE: 31225520.0.0000.5344). All participants expressed their consent to participate in the research by accepting an informed consent form available online. At the end of the research, all participants received information about the maintenance of mental health during the pandemic and, when necessary, assistance with remote psychological first aid and referrals, as previewed in the original research–intervention protocol.

## 3. Results

### 3.1. Longitudinal Change in NR Scores

The first analyses checked for change in participants who answered at least two stages. This showed mean score changes of −0.009 (95% CI = −0.071 to 0.047) between the first and second collections and −0.013 (95% CI = −0.070 to 0.053) between the second and third collections. That the confidence intervals cover no change, and that each includes the mean for the other change, indicates that, despite the small drop in score at each stage, this was neither an important nor statistically significant change.

In addition to the longitudinal effect, the variability of the change between the stages was evaluated. The mean square changes between the first and the second stages was 0.15 (95% CI = −0.011 to 0.019) and between the second and third stages, it was 0.047 (95% CI = −0.027 to 0.077). In contrast to the absence of a longitudinal effect on NR scores, there was lower variability in change between the second and third stages than between the first and second. This may indicate that people with greater mental health problems or distress were less willing to complete the last two stages of the study. These data also indicate probable distinctions between the change across participants. This result justifies subsequent exploration of effects of potential change predictors and, subsequently, the categorization of individual change using the RCI.

### 3.2. Testing Potential Predictors of NR Scores Evolution

Neither gender nor age showed a significant effect on change. Gender was related to baseline NR scores, but not to change. After evaluating these demographic predictors, potential predictors of physical exercise, home support, and professional mental health monitoring were explored.

The first variable tested was daily exercise in the baseline of the study. The data showed that the practice of exercise was significantly associated with the NR score (*p* = 0.0005) but did not significantly affect its evolution (*p* = 0.47). The data indicate that the participants who did not exercise daily had higher rates of mental distress in the first stage of the research and kept this elevated level over time.

**Figure 1:**
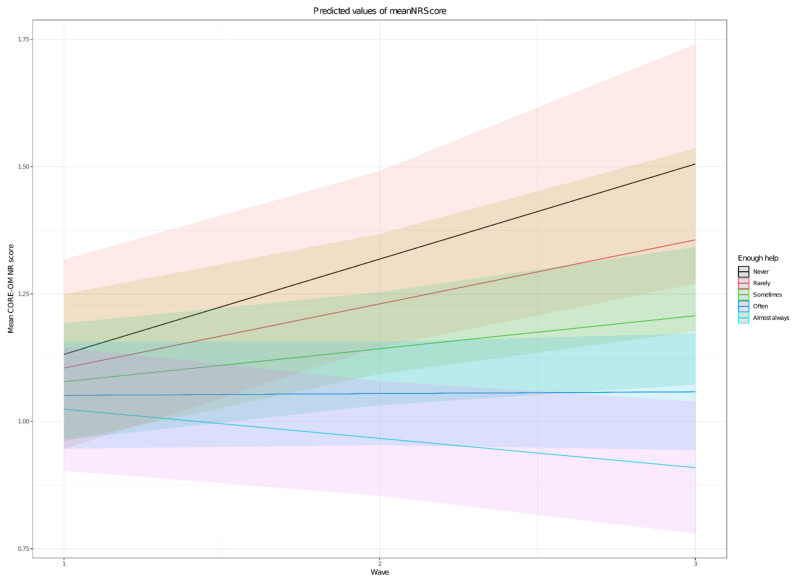
Differential effect of perceived support with household chores on change in CORE-OM NR score.

Two potential predictors addressed support received at home at baseline. Having people at home available to listen and talk was related to the general NR scores (*p* = 0.0006), but not to their longitudinal change (*p* = 0.26). On the other hand, the perceived support for domestic activities was not directly related to the baseline scores distresses (*p* = 0.40) but was related to the evolution across time (*p* = 0.001) (Figure 1).

Finally, indicators related to mental health and their association with NR scores were tested. The results of the MLM indicate that being in counseling is associated neither with mental distress in the first data collection (*p* = 0.53) nor with longitudinal change (*p* = 0.28). Very similar results were identified for psychiatric support for the NR score (*p* = 0.73) and its evolution (*p* = 0.16). The next step in the evaluation was to identify the trajectories of specific subjects and analyze some of their qualitative issues.

### 3.3. Assessment of Individual Evolution

For this analysis, only respondents from more than one stage were included, comparing them with themselves. It was considered a significant change when the participant’s data showed reliable changes in the NR. In this sense, Table 1 shows the evolution data of the participants.

From this data, it is possible to see that a similar proportion of participants showed deterioration, stability, and improvement in each of the stages. To deepen our understanding of these findings, we looked at the participants’ individual responses to the qualitative question about their main concerns. These responses were grouped into six domains: work, health, social isolation, life and personal routine, social environment, and future [23]. Many responses were complex; that is, they involved multiple domains. Some examples are presented in Table 2.

The themes included fear of contagion, problems associated with living together due to isolation, excessive domestic and work activities, and broad issues related to the economy, governmental responses, and the future. Perhaps not surprisingly, in view of the small numbers showing reliable deterioration, the qualitative categories of their comments were not very different from those obtained in our previous study with the entire group [21].

## 4. Discussion

The objective of this article was to investigate in Brazilian university workers within-participant changes in mental health during the pandemic, and the measures for their containment. The data were from three collections between the 9th and 21st weeks of the pandemic in Brazil. The response rate to the first collection was 22%, and dropped to 13.94% and 11.19%, respectively, in the second and third collections. During this period, most respondents worked remotely and voluntarily complied with the measures of social distancing so the effects of the containment measures cannot be separated from the effect of the pandemic itself, as so few did not comply with the measures. Although the rate response is lower than the ideal, this is not unexpected from a voluntary survey with no rewards for participation in very stressful times. Low response rates do not necessarily lead to response bias but happen when the variable of interest affects the decision to participate or not [34]. We acknowledge that this might be the case. Therefore, we recommend caution in interpreting and generalizing these survey results. The quantitative results showed relative stability in the levels of mental distress across the period. Despite similarity in terms of time and stage of the pandemic, the results are different from those obtained in India, which showed a great increase in mental suffering in a longitudinal study [15]. The results were also divergent from the study conducted by Canet-Juric et al. (2020) [14] in Argentina that showed small effects on indicators of depression and anxiety, and a negative effect during two weeks of lockdown. However, our findings are similar to those obtained in China [13]. In this sense, it is possible that the stability found in both studies occurs due to the balance between the advance of pandemic stressors and resilience adjustments. It is not possible to identify specific issues that justify the similarity of results between our study and the one conducted in China [13], and not with those conducted in Argentina [14] and India [15]. However, methodological (e.g., an instrument used, time of follow-up) and contextual (e.g., stage of the pandemic, the severity of distance measures, economic impact) differences may have influenced the great variability in the results of longitudinal studies during the pandemic [17].

It is noteworthy that the absence of longitudinal effects in the present study is congruent with a meta-analysis published by Prati and Mancini [17]. In this review, no significant impacts were observed during the pandemic for most mental health variables, including psychological distress. In the present study, the data collection was during the course of the pandemic, with no pre-pandemic measures; therefore, we cannot know if psychological health was or was not affected initially in the face of stressors related to the pandemic and the measures for containing it. However, the idea that the pandemic may have caused a rise in distress is suggested by the finding that mean scores in the present study are above the cut-off points for clinical groups obtained before the pandemic in the English and Spanish versions of the instrument [24,35,36].

Whatever the initial effect of the pandemic and restrictions, our findings suggest there was relative stability of the high levels of mental distress across the evaluated period. This possibility would be in line with the observation of Wang et al. (2020) [13] on the stabilization at high levels of suffering, and with the data obtained in the United Kingdom that demonstrated the effects of worsening mental struggle, comparing periods before and during the pandemic, denoting the chronicity of the response to the multiple stressors [11,12]

Despite the absence of a longitudinal effect on mental distress, the results of this study indicate a significant reduction in the variability of the level of distress of the participants in each of the moments of the study. This homogenization may also indicate a bias in which extreme participants (with positive and negative outcomes) tend not to follow all stages of the study. This issue of attrition in longitudinal studies has been little explored in the pandemic mental health literature and might be a contributor to the heterogeneity of the results of pandemic longitudinal studies [17].

A secondary objective of the study was to evaluate predictors of the longitudinal evolution of mental distress during the pandemic. The results indicate that the only variable significantly associated with the evolution of mental suffering in this period was the help received in domestic activities. This is particularly important considering the overload of these professionals who, almost all, now performed both work and domestic activities from their homes. In this sense, adjustments in work to carry out online activities and the greater support required by students may be associated with the greater overload of university workers in the period of the pandemic than for some other professions and occupations [22]. Additionally, with the loss of social support, there has been an increase in domestic and family demands that make it difficult to maintain the balance between life and work in academic contexts [37]. The demands of the pandemic have led to an increase in life–work conflicts, particularly in families with younger children [38], and this variable has been considered a predictor of disagreement and stress in family systems [39]. The qualitative stage of this study reinforces this hypothesis: participants showing reliable, psychological deterioration, like most participants, were concerned with issues beyond contagion and isolation, including other dimensions of the experience of living and working in the context of the pandemic and social detachment.

Stable predictors such as gender, age, and history of mental disorder were not related to the course of mental health in the pandemic. These variables have been reported as predictors of psychological distress during the pandemic in cross-sectional studies conducted in Brazil [9,10] and in several other countries [6,8]. Except for gender, these predictors were also associated with the level of mental distress when examined in the first stage of the present study [23]. Predictors of the evolution of mental distress in longitudinal studies are occasionally different from those identified in cross-sectional studies. This disagreement possibly occurs due to the fact that cross-sectional studies capture greater vulnerability of certain demographic groups to the emergence of psychopathology, even in periods before the pandemic (e.g., [40,41]). Additionally, there are likely differences between the immediate impact of the pandemic and its long-term effects, reinforcing the need for more longitudinal studies in many countries and different social groups.

Non-stable predictors such as exercise, people available to talk, and psychological and psychiatric consultation were not associated with the evolution of mental distress. In general, these variables had effects on mental distress in the first moment that was maintained in the other follow-up measures. Like the stable predictors, most of these variables were associated with mental distress in the cross-sectional analysis from first data collection [23]. The findings about the (psychological or psychiatric) support variable should be interpreted cautiously and in the context of the study, which carried out the data collection with the provision of mental health support to those with greater distress. That the participants knew this may have affected willingness to disclose sometimes stigmatized access to support. Hence, though the relationship found here should generalize to other surveys linked with offers of support, whether the same would be found where no support is offered cannot be known.

This study has many limitations, the main ones being sample size and unknown biases of non-participation. The sample size, though not small, reduces the precision of estimation of effects and reduces the power to detect weak effects and interactions between the predictors. As ever, non-significant effects must be interpreted with caution. Perhaps more important, is that possible biases arising from selective non-participation can, as always, not be known. Responders plus the data suggest a reduction in the variability in the response profile. However, the qualitative data of the participants’ concerns complement the conclusions of the study.

Finally, our results, both quantitative and qualitative, indicate that university workers, as presumedly most of the population, faced dramatic changes in their work–life balance during the pandemic. It is possible that the mental overload resulting from these changes, together with the fear of contagion, previous vulnerabilities, and other variables, results in further deterioration of mental health. In this sense, it is quite plausible that the support received for these additional activities (domestic) positively impacts mental health, avoiding this kind of burden.

## 5. Conclusions

This study provides important results regarding university workers, fulfilling social isolation, during the beginning of the pandemic and is supported by longitudinal, quantitative, and qualitative data. The results suggest that, after an initial negative impact, there was a relative stability of mental distress and that the support received in domestic activities minimizes psychological deterioration. New and more specific studies in this direction can provide data to assist government officials in the planning of public health actions, as well as managers with a review of possible work demands to avoid an increase in psychopathological conditions during pandemics and similar situations.

## Figures and Tables

**Table 1:** Evolution of participants in the mental health indicator considering longitudinal monitoring Reliable Change Index.

Time	Total of Participants	Deterioration N (%)	No Reliable Change *N* (%)	Improvement *N* (%)
Change wave 1 to 2	87	8 (9.2%)	70 (80.5%)	9 (10.3%)
Change wave 2 to 3	70	3 (4.3%)	62 (88.6%)	5 (7.1%)
Change wave 1 to 3	62	6 (9.7%)	48 (77.4%)	8 (12.9%)

**Table 2:** Concerns of participants who deteriorated during the 3 months of social isolation.

Vignette	Reported Concern
Vignette 1	“*The lack of direct contact with society, the difficulty in maintaining physical care routine and the distance from cultural activities, travel and entertainment*.” (Domains: social isolation; life and personal routine)
Vignette 2	“*Household chores and the uncertainty of what is to come*.” (Domains: life and personal routine and future)
Vignette 3	“*Not being able to maintain physical proximity to friends, relatives. The uncertainty about COVID, work, personal life. Suspension of some personal care, for fear of exposing myself*.” (Domains: social isolation; future; health)
Vignette 4	“*Feeling of not being able to cope with the demands; feeling of loneliness; emotional and work overload; economic situation of family members*.” (Domains: health; work; social isolation; social environment)
Vignette 5	“*Too much time in front of the PC screen*.” (Domain: work)
Vignette 6	“*Government of the country; The uncertainty about the future*.” (Domains: social environment and future)

## Data Availability

Research data are available on request from the corresponding author. Data is not public due to confidentiality.
